# *SCN9A* should not be considered an epilepsy gene; Refuting a gene–disease association

**DOI:** 10.1111/epi.18474

**Published:** 2025-06-10

**Authors:** Ismael Ghanty, Eduardo Perez‐Palma, Camilo Villaman, Daniel Stobo, Joseph Symonds, Sameer Zuberi, Dennis Lal, Andreas Brunklaus

**Affiliations:** ^1^ School of Health and Wellbeing University of Glasgow Glasgow UK; ^2^ Paediatric Neurosciences Research Group Royal Hospital for Children Glasgow UK; ^3^ Leeds Children's Hospital Leeds UK; ^4^ Leeds Institute of Medical Research University of Leeds Leeds UK; ^5^ Centro de Genética y Genómica, Facultad de Medicina Clínica Alemana Universidad del Desarrollo Santiago Chile; ^6^ West of Scotland Centre for Genomic Medicine Queen Elizabeth University Hospital Glasgow UK; ^7^ UTHealth Houston Texas USA

**Keywords:** febrile seizures, GEFS+, genetic epilepsy, SCN9A, sodium channels

## Abstract

**Objective:**

The *SCN9A* gene is primarily expressed in nociceptive pathways within the peripheral nervous system, and pathogenic variants are associated with human pain disorders. In recent years, several studies have proposed *SCN9A* as a monogenic cause of epilepsy. Our objective was to critically appraise the *SCN9A*–epilepsy gene–disease relationship.

**Methods:**

We assessed “epilepsy‐associated” *SCN9A* variants from four sources: (1) the literature up to December 2023 (*n* = 27), (2) epilepsy patients referred for genetic testing at a regional service in Glasgow, UK over a 5‐year period (*n* = 30), (3) the Human Genetics Mutation Database (*n* = 25), and (4) ClinVar (*n* = 1546). The latter two are genome‐wide variant databases, accepting submissions from genetic laboratories and research groups. We checked whether each *SCN9A* variant is present in the Genome Aggregation Database (gnomAD) V4 (a reference population database for variant interpretation), and classified its pathogenicity based on the American College of Molecular Genetics and Genomics/Association of Molecular Pathologists guidelines.

**Results:**

Only three *SCN9A* variants were classified as “likely pathogenic,” of which two were identified in healthy individuals in gnomAD. A total of 1540 of the 1546 *SCN9A* variants in ClinVar labeled as being associated with epilepsy were also reported in association with hereditary sensory and autonomic neuropathy. No further clinical data were provided in 1482 of these submissions.

**Significance:**

There is no convincing genetic evidence to support *SCN9A* as a causative epilepsy gene. As such, the inclusion of *SCN9A* in epilepsy genetic testing panels should be reassessed. Research centers and genetic testing laboratories should be rigorous and consistent in their submissions to variant databases.

**Key points**

- *SCN9A* variants are associated with pain disorders.
- Variants submitted to genomic variant databases such as ClinVar often have limited or conflicting clinical information.
- There is no genetic evidence to support *SCN9A* as a monogenic cause of autosomal‐dominant epilepsy.

## 1 INTRODUCTION

Voltage‐gated sodium (Nav) channels play a crucial role in neuronal excitability and conduction of electrical activity. Nav channels consist of a large pore‐forming α subunit that associates with one or more β subunits.^1^ Each of the nine isoforms of the sodium‐channel α subunit has a unique amino acid sequence—albeit with a sequence homology of greater than 50% in the transmembrane and extracellular domains^2^—and a distinct distribution in the central and peripheral nervous systems.^3^

Pathogenic variants in the *SCN9A* gene are known to cause human pain disorders, such as congenital insensitivity to pain and primary (inherited) erythromelalgia.^4^ Multiple studies have identified several *SCN9A* variants proposed as a monogenic cause of epileptic phenotypes, including febrile seizures, genetic epilepsy with febrile seizures plus (GEFS+), and Dravet syndrome.^5^, ^6^, ^7^, ^8^, ^9^, ^10^, ^11^, ^12^, ^13^, ^14^, ^15^, ^16^ *SCN9A* has also been posited as a genetic modifier in *SCN1A*‐related epilepsy.^5^, ^6^

Technological advances combined with the increased availability of high‐throughput next generation sequencing (gene panels, exome sequencing, and whole genome sequencing) over the past 2 decades have led to a large rise in the number of gene–disease relationships being put forward. Gene panels used for molecular diagnosis may change over time, as knowledge accumulates and new evidence emerges.

Previously, Fasham et al.^17^ disputed the link between *SCN9A* and epilepsy, presenting findings from the UK Biobank, and the Amish community in Wisconsin, USA. Our objective was to critically appraise the current evidence linking *SCN9A* variants to epilepsy. We assess *SCN9A* variants from four sources: (1) those reported in the literature; (2) those detected at a regional genetics service in Glasgow, UK over a 5‐year period; (3) the Human Genetics Mutation Database (HGMD); and (4) ClinVar. We show that there is no evidence to label *SCN9A* as a monogenic cause of epilepsy.

## 2 MATERIALS AND METHODS

### 2.1 Literature variants

We performed a PubMed search using the terms “*SCN9A*” and “epilepsy” to identify all studies published in English up to December 2023, proposing *SCN9A* variants associated with epilepsy. Twelve studies were identified, which collectively reported 27 *SCN9A* missense variants.

### 2.2 Glasgow (West of Scotland) variants

We retrospectively reviewed all *SCN9A* genetic variants detected in patients with epilepsy referred for genetic testing at the West of Scotland Genetics Service in Glasgow over a 5‐year period from January 2017 to December 2021.

### 2.3 ClinVar and HGMD

HGMD and ClinVar are the two leading genome‐wide patient variant databases. The former collates disease‐causing variants reported in the peer‐reviewed literature, whereas the latter accepts submissions from clinical testing laboratories and research groups with a variety of clinical significances. We looked at all *SCN9A* variants in these two databases, regardless of clinical phenotype. Noncoding and large‐scale chromosomal rearrangements involving multiple genes were not included. Ten variants reported in the literature were present in both ClinVar and HGMD, an additional two in ClinVar only, and six in HGMD only. These duplicates were excluded from the ClinVar and HGMD groups.

### 2.4 Variant classification and pathogenicity

The American College of Molecular Genetics and Genomics (ACMG) together with the Association of Molecular Pathologists (AMP) has published guidelines to standardize the interpretation of variant pathogenicity.^18^ This is done by weighing up different levels of evidence from four main areas: population genetics, functional, computational, and segregation/inheritance data. We performed a semiautomatic ACMG/AMP classification for each of the *SCN9A* variants in the four subgroups with the InterVar program.^19^

### 2.5 Population genetics

The Genome Aggregation Database (gnomAD) accumulates and harmonizes pre‐existing exome and genome data from a wide range of large‐scale human sequencing projects. This has been depleted from individuals known to have severe pediatric disease and their first‐degree relatives. Thus, gnomAD serves as a reference population database essential for variant interpretation. At the current sample size of gnomAD, any individual will have approximately 200 very rare exonic variants observed in gnomAD with an allele frequency < .1%, while carrying a mean of 27 unique coding variants absent from gnomAD.^20^ We checked whether each of the *SCN9A* variants are present in the gnomAD v4 database and the reported allele frequency.

## 3 RESULTS

### 3.1 Literature variants

In total, 27 *SCN9A* missense variants associated with epilepsy have been reported in the literature. Table 1 shows the associated clinical phenotypes, information on inheritance and familial segregation (where available), gnomAD allele frequencies, and our classification of the pathogenicity each of these variants.

**TABLE 1:** *SCN9A* variants reported in the literature to be associated with epilepsy.

No.	Variant	Reported clinical phenotype	*SCN9A* segregation related to epilepsy	gnomAD allele frequency, %	Experimental evidence	ACMG/AMP variant classification	References
1	c.29A>G p.(Gln10Arg)	GEFS+	Inherited from unaffected parent; present in affected sibling	1.29E‐4	Gain‐of‐function effect in DRG neurons and HEK293T cells using voltage‐clamp and current‐clamp testing respectively (Han et al. 2009^21^)	Likely benign	Cen et al. 2017^7^
2	c.184A>G p.(Ile62Val)	FS	Not stated	2.80E‐2	None	Uncertain significance	Singh et al. 2009^5^
3	c.319T>C p.(Tyr107His)	FS+	Inherited from affected parent	–	None	Uncertain significance	Banfi et al. 2020^12^
4	c.360T>G p.(Ile120Met)	Dravet Syndrome	Not stated	4.04E‐3	None	Uncertain significance	Mulley et al. 2013^6^
5	c.446C>A p.(Pro149Gln)	FS	Not stated	6.36E‐7	None	Uncertain significance	Singh et al. 2009^5^
6	c.446C>G p.(Pro149Arg)	Developmental and epileptic encephalopathy	De novo	–	None	Uncertain significance	Gowda et al. 2021^14^
7	c.554G>A p.(Arg185His)	FS+	Inherited from unaffected parent; present in affected sibling	2.80E‐3	Gain‐of‐function effect in DRG neurons using voltage‐clamp and current‐clamp testing (Han et al. 2012^22^)	Likely benign	Ma et al. 2021^15^
		Dravet syndrome	Not stated				Mulley et al. 2013^6^
		Developmental and epileptic encephalopathy	De novo				Gowda et al. 2021^14^
8	c.796C>A p.(Leu266Met)	GEFS+	Inherited from affected parent	3.99E‐3	None	Uncertain significance	Mulley et al. 2013^6^
9	c.980G>A p.(Gly327Glu)	SeLECTS	Inherited from unaffected parent; present in affected twin sibling	4.57E‐2	None	Uncertain significance	Liu et al. 2019^9^
		GEFS+	Inherited from affected parent				Yang et al. 2018^8^
10	c.1285C>T p.(Arg429Cys)	FS+	Inherited from unaffected parent	8.06E‐3	None	Uncertain significance	Ding et al. 2019^10^
11	c.1324G>A p.(Ala442Thr)	GEFS+	Inherited from unaffected parent; present in affected uncle	9.44E‐2	None	Likely benign	Ding et al. 2019^10^
12	c.1469G>A p.(Ser490Asn)	FS	Not stated	1.52E‐2	None	Likely benign	Singh et al. 2009^5^
13	c.1555G>A p.(Glu519Lys)	Dravet syndrome	Inherited from parent of unknown phenotype	4.50E‐4	None	Uncertain significance	Singh et al. 2009^5^
14	c.1828C>A p.(Pro610Thr)	Developmental and epileptic encephalopathy	De novo	.026	None	Likely benign	Gowda et al. 2021^14^
15	c.1844A>G p.(Asn615Ser)	Developmental and epileptic encephalopathy	De novo	.0000112	None	Uncertain significance	Gowda et al. 2021^14^
16	c.1921A>T p.(Asn641Tyr)	FS, AFS, TLE	Autosomal dominant variant in the Amish community in Wisconsin	2.42E‐2	Gain‐of‐function effect in HEK293T cells using voltage‐clamp testing (Zhang et. 2020^23^)	Uncertain significance	Singh et al. 2009^5^
17	c.1964A>G p.(Lys655Arg)	GEFS+, KBG syndrome	Inherited from unaffected parent; present in affected sibling	1.95E‐3	Gain‐of‐function effect in HEK293T cells using voltage‐clamp testing (Zhang et. 2020^23^)	Uncertain significance	Alves et al. 2019^11^
		FS	Not stated				Singh et al. 2009^5^
		Dravet syndrome	Inherited from parent of unknown phenotype				Singh et al. 2009^5^
18	c.2215A>G p.(Ile739Val)	FS	Inherited from parent of unknown phenotype	2.45E‐3	None	Uncertain significance	Singh et al. 2009^5^
19	c.2325C>G p.(Ile775Met)	FS	Inherited from unaffected parent	–	None	Uncertain significance	Ding et al. 2019^10^
20	c.3488T>C p.(Trp1150Arg)	FS	Not stated	8.81E‐1	Gain‐of‐function effect in HEK293T cells using voltage‐clamp testing	Likely benign	Zhang et al. 2020^13^
21	c.3478G>C p.(Glu1160Gln)	Dravet syndrome	Inherited from unaffected parent	–	None	Uncertain significance	Singh et al. 2009^5^
22	c.3832C>T p.(Leu1278Phe)	Developmental and epileptic encephalopathy	De novo	–	None	Uncertain significance	Gowda et al. 2021^14^
23	c.4664T>C p.(Leu1555Pro)	Dravet syndrome	Not stated	–	None	Uncertain significance	Mulley et al. 2013^6^
24	c.4702A>C p.(Asn1568His)	Juvenile myoclonic epilepsy	De novo	1.64E‐2	None	Uncertain significance	Albaradie et al. 2021^16^
25	c.4999A>G p.(Met1667Val)	Developmental and epileptic encephalopathy	De novo	–	None	Uncertain significance	Gowda et al. 2021^14^
26	c.5734G>T p.(Asp1924Tyr)	Developmental and epileptic encephalopathy	De novo	–	None	Uncertain Significance	Gowda et al. 2021^14^
27	c.5873A>G p.(Tyr1958Cys)	GEFS+	Inherited from unaffected parent; present in affected aunt and in grandmother (unknown phenotype)	8.06E‐3	None	Uncertain significance	Zhang et al. 2020^13^

Abbreviations: ACMG, American College of Molecular Genetics and Genomics; AFS, afebrile seizures; AMP, Association of Molecular Pathologists; DRG, dorsal root ganglion; FS, febrile seizures; FS+, febrile seizures plus; GEFS+, genetic epilepsy with febrile seizures plus; KBG syndrome, KBG syndrome; SeLECTS, self‐limited epilepsy with centrotemporal spikes; TLE, temporal lobe epilepsy.

**FIGURE 1:**
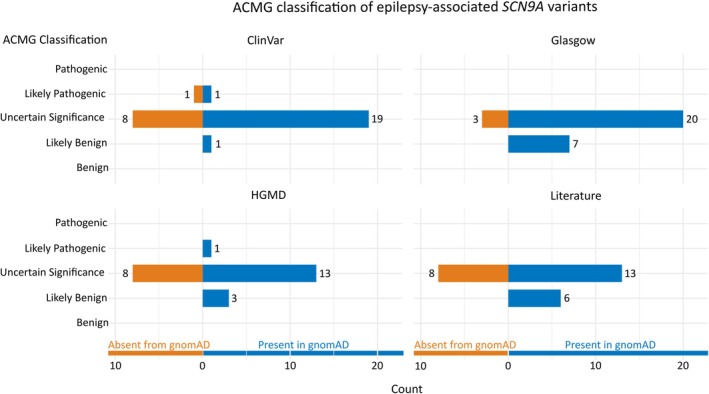
Pathogenicity of epilepsy‐associated *SCN9A* variants. Horizontal stacked bar charts display the pathogenicity of each *SCN9A* variant (as per the American College of Molecular Genetics and Genomics guidelines) from the four sources, namely, literature, Glasgow (West of Scotland), Human Genetics Mutation Database (HGMD), and ClinVar. Variants present in healthy individuals in the Genome Aggregation Database (gnomAD) are highlighted in a different color. ACMG, American College of Molecular Genetics and Genomics.

Six variants are predicted to be “likely benign,” whereas the remaining 21 are of uncertain significance (Figure 1). Nineteen variants are observed in unaffected individuals in gnomAD, including six variants that have been identified in homozygous individuals.

### 3.2 Glasgow (West of Scotland) variants

Thirty missense *SCN9A* variants were detected in 55 patients with epilepsy referred for genetic testing in the West of Scotland over the 5‐year period. Seven of these variants are classified as likely benign, and the remaining 23 are of uncertain significance (Figure 1). Twenty‐seven variants are present in gnomAD, of which seven have been identified in homozygous individuals.

### 3.3 HGMD variants

There are 190 *SCN9A* variants in HGMD, of which 25 were reported to be associated with epilepsy.

Figure 1 shows our interpretation of the clinical significance of these variants. Only one variant was predicted to be “likely pathogenic” (c.3476_3477delTA), although that variant was also present in gnomAD.

### 3.4 ClinVar variants

**FIGURE 2:**
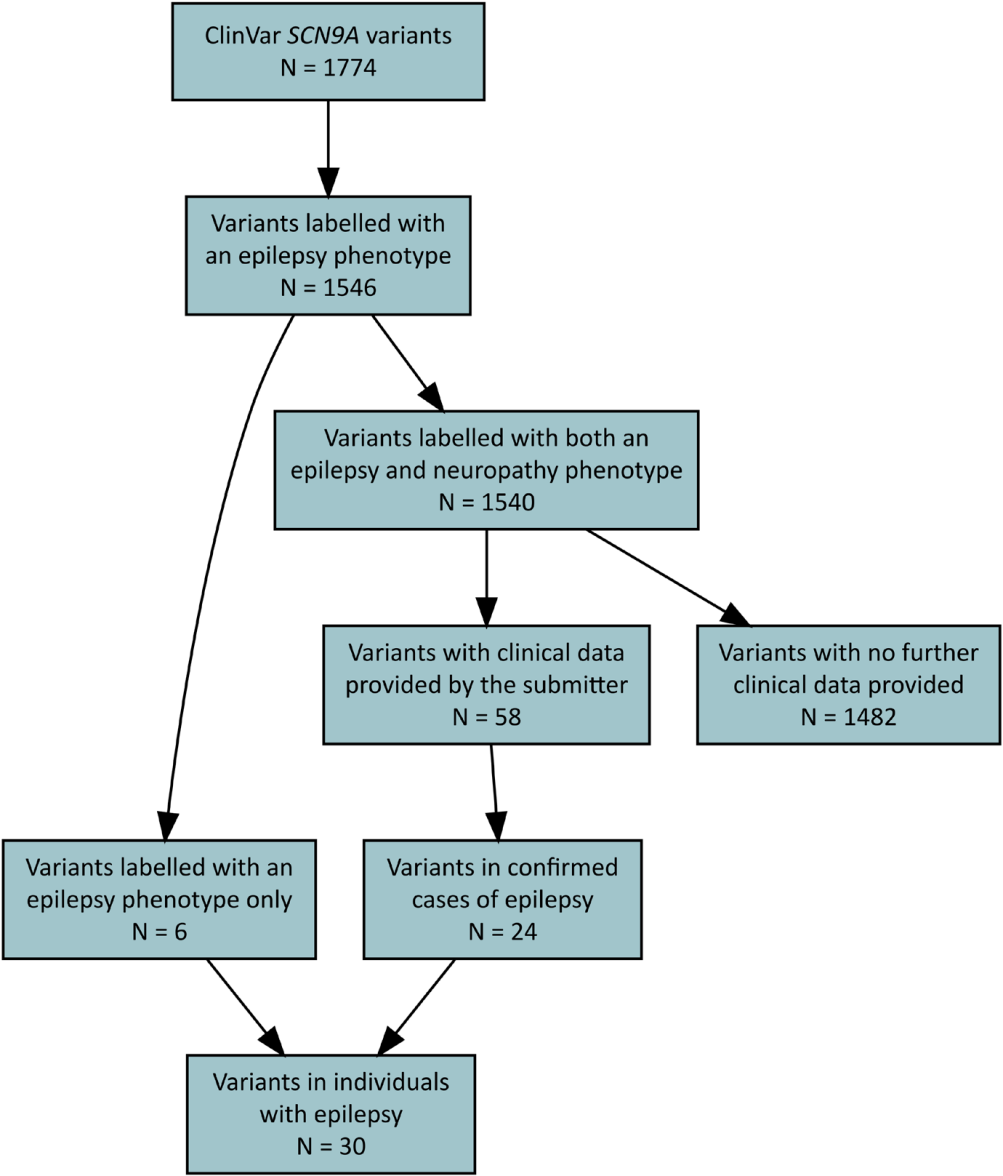
Flowchart of ClinVar *SCN9A* variants.

There were 1774 *SCN9A* variants in ClinVar in total, of which 1546 were labeled as being associated with epilepsy. Whereas only six variants (.4%) were reported in epilepsy alone, each of the remaining 1540 variants were also reported to be associated with hereditary sensory and autonomic neuropathy. Further clinical data were provided in only 58 of these submissions, of which 24 had an epilepsy phenotype. Thus, from an initial 1774 count, we ended up with 30 *SCN9A* variants, as summarized in Figure 2. Our classification of these variants is shown in Figure 1D. Two variants are predicted to be likely pathogenic: p.Trp1161Ter and p.Asp1959Ala. The former is present in gnomAD, whereas the latter is not.

### 3.5 Pathogenic *SCN9*A variants absent from gnomAD

**FIGURE 3:**
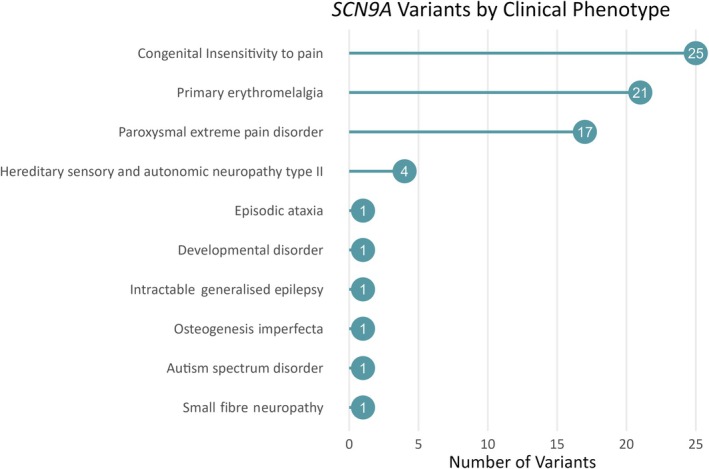
Clinical phenotypes in “likely pathogenic” and “pathogenic” *SCN9A* variants not present in the Genome Aggregation Database.

Of the *SCN9A* variants associated with any disease (epilepsy as well as nonepilepsy phenotypes) and predicted to be likely pathogenic or pathogenic, we looked specifically at those not observed in gnomAD, that is, absent from the general population. There were 63 such variants in HGMD and 57 in ClinVar. Eleven variants were reported in both databases, giving a total of 109 variants. Eighty‐eight variants had a defined clinical phenotype provided by the submitter. Figure 3 shows the distribution of clinical phenotypes observed with these variants. Only one variant, p.Asp1959Ala, has been reported to be associated with epilepsy.

## 4 DISCUSSION

The *SCN9A* gene is primarily expressed within sensory and sympathetic ganglion neurons^24^, ^25^ and epidermal free nerve endings.^26^ Pathogenic variants in *SCN9A* cause a spectrum of familial pain disorders, consistent with their expression in the peripheral nervous system and involvement in nociceptive pathways.^4^ *SCN9A* missense variants were first observed in conditions with excess pain such as primary erythromelalgia^27^ and paroxysmal extreme pain disorders.^28^ These conditions have an autosomal dominant pattern of inheritance. Functional studies of these mutations using voltage‐clamp and patch‐clamp techniques have shown gain‐of‐function effects at the level of the channel or expressed neuron.^4^, ^29^

Conversely, congenital insensitivity to pain, characterized by an absence or loss of pain, is an autosomal recessive disorder caused by loss‐of‐function Nav1.7 variants, including nonsense, frameshift, and splice site variants.^30^, ^31^ Affected individuals harbor homozygous or compound heterozygous variants. Thus, parents (and offspring) of an affected individual are obligate heterozygotes, that is, carriers of a pathogenic variant, but are asymptomatic.

None of the 27 variants reported in the literature in association with epilepsy would be classified as pathogenic based on the ACMG/AMP criteria (six benign, 21 of uncertain significance). Nineteen variants are observed at a high allele frequency in gnomAD (Table 1), which is not in keeping with pathogenicity for epilepsy. Of the remaining eight variants, two were inherited from an unaffected parent (p.[Ile775Met] and p.[Glu1160Gln]), and a further variant had no stated inheritance information (p.[Leu1555Pro]). Another variant, p.(Tyr107His), was reported by Banfi et al.^12^ in an individual with febrile seizures plus, neurodevelopmental delay, and autism spectrum disorder. This heterozygous variant was inherited from the proband's mother, also affected by epilepsy. Genetic testing, however, also revealed a de novo duplication of 1.3 Mb in 8p21.3, a chromosomal region containing genes involved in neuronal development, as well another heterozygous genetic variant in *AKAP9* inherited from his mother. Thus, there exists a plausible alternate molecular cause for this child's disease. The remaining four variants, (p.[Pro149Arg], p.[Leu1278Phe], p.[Met1667Val], and p.[Asp1924Tyr]), are all de novo variants identified by Gowda et al.^14^ in a retrospective study of children with developmental and epileptic encephalopathies in Bangalore, India. Each of these four variants are classified as variants of uncertain significance. There is insufficient available information to evaluate these variants further. In total, Gowda et al.^14^ discovered eight *SCN9A* variants in their cohort of 50 children. The authors only reported one of these variants, p.(Pro610Thr), as pathogenic. However, this variant is present in gnomAD at a high allele frequency, including in 674 homozygous individuals. Therefore, we would classify it as likely benign.

Cosegregation of a genetic variant with disease in similarly affected members of a family is an observation that could support a gene–disease relationship. Conversely, a lack of segregation with disease, for example, identifying a variant in some, but not all, affected family members and/or identifying that same variant in unaffected individuals, would suggest that the variant is not disease‐causing. Segregation studies were only performed for 14 *SCN9A* variants reported in the literature, revealing that nine were inherited from an unaffected parent. However, segregation analysis was usually confined within nuclear, single‐generation families. The one notable exception is the p.(Asn641Tyr) variant, which segregated with disease in 21 individuals with febrile and afebrile seizures in a large family in Utah, USA.^5^ However, this variant is carried by unaffected individuals in gnomAD and has also been identified at high frequency within the Wisconsin Amish community in individuals with no history of seizures.^17^ Owing to stronger genetic drift, rare and ultrarare genetic variants can become highly enriched in certain regions (e.g., the Shetland Islands) or communities (e.g., the Amish) owing to geographical isolation, population bottlenecks, or endogamy.^17^, ^32^ These variants can be deleterious and expected to cause disease, or benign and of no clinical significance.

It is worth noting that there is significant clinical heterogeneity in the epileptic phenotypes described in the literature in association with *SCN9A*. Three of the published variants have been identified in more than one individual. Despite having the same *SCN9A* variant, each individual had a different epilepsy diagnosis. The first variant, p.(Arg185His), was detected in three patients with febrile seizures plus, Dravet syndrome, and an unspecified developmental and epileptic encephalopathy, respectively.^14^, ^15^, ^16^ This variant has also been found in an individual with small fiber neuropathy. Another variant, p.(Gly327Glu), has been described in association with GEFS+ and self‐limited epilepsy with centrotemporal spikes.^8^, ^9^ In both cases, the variants were inherited from a parent (affected in the latter case, unaffected in the former). Finally, p.(Lys655Arg) was discovered in separate patients with febrile seizures, GEFS+, and Dravet syndrome, respectively.^5^, ^11^

The functional effect of five variants reported in the literature was tested experimentally (Table 1).^11^, ^15^, ^20^, ^22^ Using voltage‐clamp and/or current‐clamp techniques, all five were shown to have overall gain‐of‐function effects. Two of these variants (p.[Gln10Arg] and p.[Arg185His]) have been detected in individuals with primary erythromelalgia and small fiber neuropathy, respectively. The remaining three (p.[Asn641Tyr], p.[Lys655Arg], p.[Trp1150Arg]) are all present in gnomAD and not rare enough in the general population to be consistent with disease.

There was one “likely pathogenic” *SCN9A* variant on HGMD, reported in epilepsy. No further information is provided regarding phenotype, family history of epilepsy, or segregation analysis. Therefore, it is difficult to speculate further on this variant's association with epilepsy. There were two “likely pathogenic” *SCN9A* variants on ClinVar, associated with epilepsy. The first, p.Trp1161Ter, was detected in a patient with seizures and neurodevelopmental delay, and reported by Laboratorio de Genetica e Diagnostico Molecular at the Hospital Israelita Albert Einstein in Brazil. There was no family history or segregation data provided. This variant was also reported to ClinVar by the Illumina Clinical Services Laboratory, after being detected in a healthy population as part of a predisposition screen. Furthermore, this variant has been reported 26 times in gnomAD in healthy individuals. The second variant, p.Asp1959Ala, was submitted to ClinVar by Invitae, a genetic testing company, in December 2017. It was detected in an individual with intractable generalized epilepsy and static encephalopathy and was de novo. This variant is not present in population databases. The lack of additional clinical information makes it difficult to further appraise this variant.

Figure 3 serves as a timely reminder of where the current evidence lies regarding the disease‐causing nature of the *SCN9A* gene. Ninety‐three percent of pathogenic *SCN9A* variants absent from the healthy population cause familial human pain disorders.

Our analysis of the *SCN9A* variants on ClinVar uncovered an important issue with the way variants are reported to and displayed on ClinVar. A total of 1540 *SCN9A* variants were presented on ClinVar as having an association with both epilepsy and hereditary sensory and autonomic neuropathy. These include 1537 variants submitted by Invitae, each with the aforementioned dual association. We note that the majority of these submissions have no additional clinical information provided, and there is no supporting evidence to suggest that each of these variants has been detected either in individuals with both epilepsy and neuropathy or in separate individuals with either epilepsy or neuropathy. Similarly, Ambry Genetics, another testing laboratory, has reported 248 *SCN9A* variants to ClinVar, each with the phenotype “inborn genetic disease.” This designation is of limited utility to clinicians or geneticists.

ClinVar has developed a four‐star rating system describing the review status of each variant submitted to its database. By default, submissions have the label “single submitter—criteria not provided” and no stars. Stars are gained if “assertion criteria” are provided, if there are multiple submitters who agree on the variant classification, or if the classification has been approved by an “expert panel.”^33^, ^34^ The *SCN9A* variants reported to ClinVar by Invitae, where Invitae was the only submitter, have a one‐star rating. This reflects the assertion criteria provided by Invitae, which includes the variant classification system used, and the criteria used to class each variant. ClinVar recommends that expert panels consist of clinicians, geneticists, clinical laboratory diagnosticians, and researchers with experience relevant to the gene–disease relationship in question. However, only a tiny fraction of genes associated with a Mendelian disorder have had variant classifications reviewed by an expert panel on ClinVar.^34^, ^35^ Furthermore, the phenotype provided by the submitter is not reviewed by ClinVar.

ClinGen is a National Institutes of Health‐funded resource that has developed a framework to standardize the approach to determining the clinical validity of gene–disease pairs in Mendelian disorders.^36^ Although the Epilepsy Gene Curation Expert Panel previously classified the validity of the *SCN9A*–epilepsy relationship on ClinGen as “limited,” since September 2021 it refutes this relationship. Our study, which included an analysis of *SCN9A* variants from different sources, provides transparent and novel evidence to invalidate the relationship between *SCN9A* and epilepsy. The high minor allele frequency in gnomAD of proposed “epilepsy‐associated” *SCN9A* variants and the lack of multigenerational segregation analysis of phenotype and genotype do not support any causative role between *SCN9A* and epilepsy. It is also important to highlight that whereas a genetic variant may be classified as pathogenic for a disease in which there is an established causative role, this assertion cannot be applied for that same variant with another unrelated disease group. Therefore, there is a real possibility of patients with epilepsy being mislabeled with a genetic diagnosis if a pathogenic *SCN9A* variant is discovered.

Nonetheless, research centers and diagnostic laboratories continue to report *SCN9A* variants in association with epilepsy in the literature or in variant databases. There were submissions of “likely pathogenic” (c.596+1G>T) and “pathogenic” (3780G>A[p.Trp1260Ter]) *SCN9A* epilepsy variants to ClinVar as recently as January 2024. It is clear that there is still uncertainty within the scientific community regarding the *SCN9A*–epilepsy gene–disease relationship. Gene–disease association classifications may also differ between organizations. For example, Invitae still asserts (as of November 2023) that there is strong evidence of *SCN9A* variants being a cause of GEFS+. A study like ours is rarely undertaken and would not usually result in a high‐impact publication or be disseminated to a wider audience compared to one presenting a novel causative gene. However, it is crucial to comment on and address the lack of transparency in existing variant databases such as ClinVar. We should also highlight that *SCN9A* is not the first or only candidate gene with a disputed association with epilepsy. *CACNB4*, *CLCN2*, *MAGI2*, and *SRPX2* variants have all been proposed as causes of epilepsy. Common issues prevailing in the relevant studies include the reporting of variants present in gnomAD at high allele frequencies, a lack of cosegregation with disease, and the presence of plausible alternate genetic causes in the individuals tested. In a study of 6994 patients with epilepsy, Heyne et al.^37^ showed similar frequencies of ultrarare variants in these four genes in epilepsy cases and controls. Another example is a study published in the *New England Journal of Medicine* that identified *ICK* genetic variants in 7% of a cohort of 310 individuals with juvenile myoclonic epilepsy.^38^ However, these findings could not be replicated in a larger case–control analysis.^39^

The use of targeted disease‐specific genetic testing panels including genes with evidence of disease causation helps to improve diagnostic sensitivity while reducing the findings of variants of uncertain significance that would follow whole exome or whole genome sequencing.^40^ However, there is no standardized framework determining which genes are included in disease‐specific genetic testing panels, and therefore, these may vary significantly between laboratories. Genes may be tested because of specific research interests and despite a lack of relevant evidence to support pathogenicity.^41^ The inclusion of incongruous genes can lead to incorrect diagnoses and inappropriate treatment being started. We propose that the *SCN9A* gene should not be included in epilepsy gene panels.

This study has limitations. We were not able to verify the clinical phenotypes associated with the *SCN9A* variants on ClinVar and HGMD, relying instead on the details provided by the submitter. In several cases, the lack or incompleteness of clinical and genetic data precludes a more comprehensive analysis.

## 5 CONCLUSIONS

In conclusion, there is no convincing genetic evidence to support *SCN9A* as a monogenic cause of autosomal‐dominant epilepsy. As such, the inclusion of *SCN9A* in epilepsy genetic testing panels should be reassessed. Finally, genetic testing laboratories should be rigorous and consistent in their submissions to variant databases. Although this study focused on *SCN9A* and epilepsy, the lessons learned from our investigation can apply more broadly to other gene–disease relationships, especially when interpreting the significance of novel genetic variants.

## AUTHOR CONTRIBUTIONS

**Ismael Ghanty:** Study conception and design; data analysis and interpretation of results; draft manuscript preparation; reviewing and editing. **Eduardo Perez‐Palma:** Study conception and design; data analysis and interpretation of results; draft manuscript preparation; reviewing and editing. **Camilo Villaman:** Study conception and design; interpretation of results; reviewing and editing. **Daniel Stobo:** Study conception and design; interpretation of results; reviewing and editing. **Joseph Symonds:** Study conception and design; interpretation of results; reviewing and editing. **Sameer Zuberi:** Study conception and design; interpretation of results; reviewing and editing. **Dennis Lal:** Study conception and design; data analysis and interpretation of results; reviewing and editing. **Andreas Brunklaus:** Project supervision and planning; study conception and design; data analysis and interpretation of results; draft manuscript preparation; reviewing and editing.

## FUNDING INFORMATION

This research received no specific grant from any funding agency in the public, commercial, or not‐for‐profit sectors.

## CONFLICT OF INTEREST STATEMENT

None of the authors has any conflict of interest to disclose. We confirm that we have read the Journal's position on issues involved in ethical publication and affirm that this report is consistent with those guidelines.

## Data Availability

Some of the data that support the findings of this article are openly available from ClinVar and HGMD, respectively. The Glasgow (West of Scotland) dataset is not publicly available due to privacy or ethical restrictions but is available upon reasonable request from the corresponding author.
